# Clinical Significance of Soluble CD26 in Malignant Pleural Mesothelioma

**DOI:** 10.1371/journal.pone.0115647

**Published:** 2014-12-19

**Authors:** Nobukazu Fujimoto, Kei Ohnuma, Keisuke Aoe, Osamu Hosono, Taketo Yamada, Takumi Kishimoto, Chikao Morimoto

**Affiliations:** 1 Department of Medical Oncology, Okayama Rosai Hospital, Okayama, Japan; 2 Department of Therapy Development and Innovation for Immune Disorders and Cancers, Graduate School of Medicine, Juntendo University, Hongo, Bunkyo-ku, Tokyo, Japan; 3 Department of Medical Oncology and Clinical Research, National Hospital Organization Yamaguchi-Ube Medical Center, Ube, Yamaguchi, Japan; 4 Department of Pathology, Keio University school of Medicine, Tokyo, Japan; 5 Department of Internal Medicine, Okayama Rosai Hospital, Okayama, Japan; University of Central Florida, United States of America

## Abstract

There is no established single diagnostic marker for malignant pleural mesothelioma (MPM). CD26 is a 110 kDa, multifunctional, membrane-bound glycoprotein that has dipeptidyl peptidase IV (DPPIV) enzyme activity. The aim of this study was to evaluate the clinical significance of soluble CD26 (sCD26) in patients with MPM. The study included 80 MPM patients, 79 subjects with past asbestos exposure (SPE), and 134 patients with other benign pleural diseases (OPD) that were included as a control group. sCD26 levels and DPPIV activity in serum and/or pleural fluid were determined using an ELISA kit. Serum sCD26 levels and DPPIV enzyme activity in patients with MPM were significantly decreased compared with those in the SPE group (P = 0.000). The level of serum sCD26 was significantly decreased in patients with advanced stages of MPM compared with those with earlier stages (P = 0.047). The median OS of patients with MPM who had higher DPPIV enzyme activity was significantly longer than that of those with lower DPPIV enzyme activity (P = 0.032). The sCD26 levels in the pleural fluid of MPM patients with an epithelioid subtype were significantly increased compared with the OPD cohort (P = 0.012). Moreover, DPPIV enzyme activity in the pleural fluid of patients with MPM with an epithelioid subtype were significantly increased compared with those in the OPD cohort (P = 0.009). Patients with MPM who had lower specific DPPIV activity, determined as DPPIV/sCD26, showed significantly prolonged survival compared with those with higher specific DPPIV activity (P = 0.028). Serum sCD26 and DPPIV enzyme activity appear to be useful biomarkers for differentiating patients with MPM from SPE. The sCD26 levels or DPPIV enzyme activity in pleural fluid appear to be biomarkers in patients with an epithelioid subtype of MPM. DPPIV activity in serum or pleural fluid appears to be predictive for the prognosis of patients with MPM.

## Introduction

Malignant pleural mesothelioma (MPM) is an aggressive malignancy arising from the mesothelial cells lining the pleura [1]. It is generally associated with a history of asbestos exposure [2] and has a very poor prognosis [3]. Once rare, the incidence of MPM has increased in industrialized nations including Japan and the United States as a result of past wide-spread exposure to asbestos [4]. The incidence of MPM is predicted to increase in the next decades, especially in developing countries where asbestos has not yet been banned [1], [4], [5]. Treatment for MPM includes surgery, radiotherapy, and/or systemic chemotherapy, but the effectiveness of these interventions is limited. Therefore, novel strategies for early diagnosis and screening of people with past asbestos exposure who are at high risk are urgently needed to improve the outcome.

There is presently no established single diagnostic marker of clinical significance for MPM. Soluble mesothelin-related peptides (SMRP) appear promising for differentiating MPM from lung cancer (LC) [6], [7]. Recently, Shiomi et al reported that N-ERC/mesothelin may be a useful marker for diagnosing MPM [8]. Pass et al reported that plasma fibulin-3 levels could distinguish healthy persons with exposure to asbestos from patients with MPM [9]. However, these markers have not yet been established for use in clinical practice.

CD26 is a 110 kDa, multifunctional, membrane-bound glycoprotein, with dipeptidyl peptidase IV (DPPIV) enzyme activity in its extracellular domain [10] and is critical in T-cell biology as a marker of T-cell activation [11]–[13]. CD26 has an important but complex function in tumor behavior. Its biological effect depends on the tumor type and microenvironment. It is a marker of aggressive disease for certain subsets of T-cell non-Hodgkin's lymphomas/leukemias where expression of CD26 on T-lymphoblastic lymphomas/acute lymphoblastic leukemia cells is associated with a worse outcome compared with CD26-negative tumors [14]. CD26 is also expressed at high levels on renal carcinoma cells [15]–[17]. Recently, we showed that CD26 is preferentially expressed on malignant mesothelioma cells, but not on normal mesothelial cells. More importantly, humanized anti-CD26 antibody inhibited the growth of malignant mesothelioma cells and induced long-term survival of tumor-transplanted SCID mice [18]. More recently, we planned a treatment outcome prediction study and showed that CD26 membrane expression on MPM cells was closely correlated with responsiveness of the disease to chemotherapy [19]. All these findings suggest that CD26 would be a significant biomarker of MPM.

In the current study, we determined soluble CD26 (sCD26) and DPPIV enzyme activity in the serum and pleural fluid of patients with MPM. The aim of this study was to evaluate the clinical significance of sCD26 as a screening, early diagnosis, and/or prognostic marker of MPM.

## Materials and Methods

### Subjects

The study included 80 MPM patients diagnosed and treated at Okayama Rosai Hospital and National Hospital Organization Yamaguchi-Ube Medical Center between 1998 and 2013. Histological sections from the patients with mesothelioma were examined and classified by immunohistochemistry as epithelioid, biphasic, or sarcomatous subtypes according to the World Health Organization histological classification [20]. Clinical stage was determined according to the criteria of the International Mesothelioma Interest Group TNM staging system for MPM [21]. Seventy-nine subjects with past asbestos exposure (SPE) and pleural plaques seen on chest computed tomography, and 134 patients with other benign pleural diseases (OPD) as a control group were also included. Portions of MPM and OPD were previously reported in our previous studies of SMRP [7] and hyaluronic acid determination [22]. Written informed consent was obtained from all patients.

### Measurement of sCD26 or DPPIV Enzyme Activity

Serum samples were collected from 41 (29 epithelioid, 4 sarcomatous, and 8 biphasic) out of 80 patients with MPM, and from all those with SPE. Pleural fluid samples were collected from 65 (43 epithelioid, 15 biphasic, 7 sarcomatous) out of 80 patients with MPM, and all patients with OPD. The current study was initiated by determining sCD26 in pleural fluid. Subsequently, we added the analyses of serum sCD26. That is why there was lost data of MPM patients. For measurement of serum sCD26 levels or DPPIV enzyme activity, the serum or fluid samples were collected and stored at −80°C until measurement. Methods for measuring sCD26 and DPPIV enzyme activity were developed in our laboratory and have been described in detail elsewhere [23].

### Measurement of SMRP

SMRP was measured by the chemiluminescent enzyme immunoassay (CLEIA) (Fujirebio Diagnostics. Malven, USA) based on 2-step sandwich method described in detail elsewhere [7].

### Statistical Analysis

The results are shown as numbers (n), medians ± standard deviation (SD), or medians and interquartile range. Differences in means for laboratory data were analyzed by analysis of variance for multiple comparisons or two-tailed Student's t test for group comparisons. Areas under the receiver operating characteristic (ROC) curves (AUCs) were calculated using standard techniques. Overall survival (OS) of patients with MPM was defined as the time from the day of diagnosis to the date of death or last follow-up. The proportion of survival and 95% confidence intervals (CI) were determined based on the Kaplan-Meyer method. Correlation was calculated as Pearson product-moment correlation coefficient. Statistical calculations were performed using the IBM SPSS Advanced Statistics19 (IBM Japan, Tokyo, Japan). All reported P values are two-sided. A level of P <0.05 was accepted as statistically significant.

### Study Approval

Human study protocols were approved by the Ethics Committees at Okayama Rosai Hospital, National Hospital Organization Yamaguchi-Ube Medical Center, and Juntendo University. All studies on human subjects were carried out according to the principles set out in the Declaration of Helsinki.

## Results

### Demographic and Clinical Characteristics of the Study Populations

Of the 80 patients with MPM, the median (years ± SD) age was 69 (±9.13) years, and 75 were males and 5 were females. An occupational history of asbestos exposure was indicated in 75 patients and the median (± SD) duration of asbestos exposure was 34 (±13.75) years. Of the group of 79 SPE, the median (± SD) age was 66 (±5.50) years, 78 were males and 1 was female, and the median duration of asbestos exposure (± SD) was 23 (±12.87) years. Of the 134 OPD patients, the median (± SD) age was 76 (±10.34) years and 122 were males and 12 were females. The median age was significantly higher (P = 0.000) and there were significantly more female patients (P = 0.000) in OPD patients than in other groups.

### Serum sCD26 and DPPIV Activity of Each Cohort

**Figure 1:**
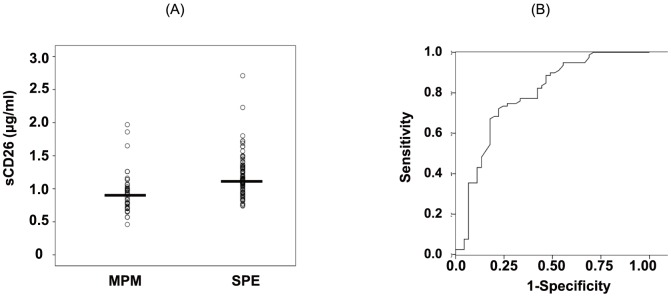
Comparison of serum soluble CD26 (sCD26) levels. (A) Comparison of serum sCD26 levels in patients with malignant pleural mesothelioma (MPM) or subjects with past asbestos exposure (SPE). Each dot indicates an individual value and the horizontal bar indicates the median value. (B) Receiver operating curve analysis of sCD26 levels according to the differentiation between patients with MPM and SPE.

The median and interquartile range values of the serum and pleural fluid sCD26 levels and DPPIV enzyme activity are shown in S1 Table. To determine whether or not the serum levels of sCD26 or DPPIV enzyme activity were biomarkers among MPM patients, we first analyzed the differences in the serum levels of sCD26 or DPPIV enzyme activity between the MPM and SPE cohorts. As shown in Fig. 1A, serum sCD26 levels in patients with MPM were significantly decreased compared with the SPE group (P = 0.000). To further clarify the usefulness of serum sCD26 levels for differentiating MPM from SPE, we performed a ROC analysis. The AUC value for the differential diagnosis between these 2 groups was 0.775 (95% CI, 0.682–0.868) (Fig. 1B). Based on a cutoff value of 1.00 µg/ml, the sensitivity was 74.7% and the specificity was 71.4% (Fig. 1B).

**Figure 2:**
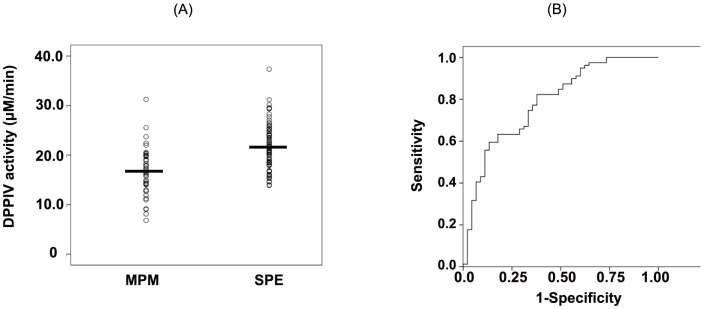
Comparison of serum dipeptidyl peptidase IV (DPPIV) enzyme activity levels. (A) Comparison of serum DPPIV enzyme activity levels in the sera of patients with malignant pleural mesothelioma (MPM) or subjects with past asbestos exposure (SPE). Each dot indicates an individual value and the horizontal bar indicates the median value. (B) Receiver operating curve analysis of serum DPPIV enzyme activity according to the differentiation between patients with MPM and SPE.

sCD26 possesses DPPIV enzyme activity, which cleaves cytokines, chemokines, or peptide hormones to regulate their actions [10]. We examined the serum DPPIV enzyme activity to determine its usefulness as a biomarker for differentiating MPM from SPE. As shown in Fig. 2A, serum DPPIV enzyme activity is significantly decreased in patients with MPM compared with those with SPE (P = 0.000). The ROC curve shows that the AUC value for the differential diagnosis of these 2 groups was 0.778 (95% CI, 0.690-0.865). Based on a cutoff value of 17.0 µM/min, the sensitivity was 52.4% and the specificity was 82.3% (Fig. 2B). Taken together, these results indicate that serum DPPIV enzyme activity, as well as serum sCD26 levels, appear to be useful biomarkers for differentiating MPM from the SPE group.

### Analysis of Serum sCD26 or DPPIV Activity Among Patients with MPM

**Figure 3:**
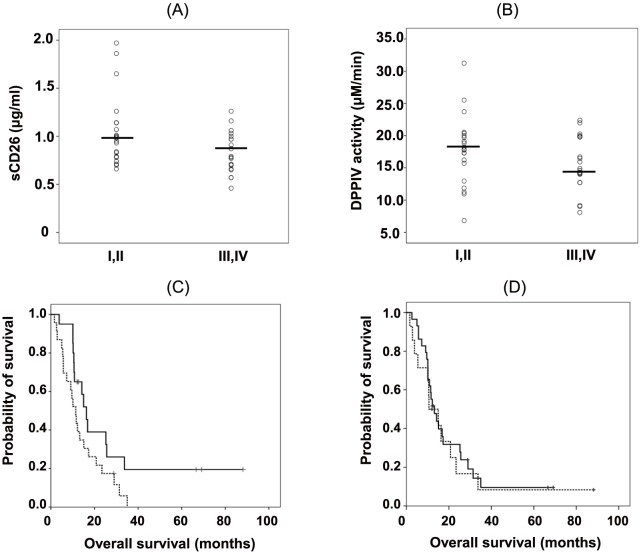
Serum sCD26 levels or DPPIV enzyme activity according to clinical stage and outcomes. (A) Comparison of levels of serum sCD26 levels and (B) DPPIV enzyme activity among patients with MPM according to clinical stage. The International Mesothelioma Interest Group TNM staging system for MPM was used to determine stage I–IV MPM. The mean values are indicated by horizontal lines. Each dot indicates an individual value and the horizontal bars indicate the median value. (C) Overall survival (OS) in patients with MPM according to those with higher serum sCD26 values (≥1.00 µg/ml, solid line) and lower serum sCD26 values (<1.00 µg/ml, dashed line). (D) OS in patients with MPM according to those with higher (≥17.0 µM/min, solid line) and lower (<17.0 µM/min, dashed line) serum DPPIV enzyme activity.

As shown above, the serum sCD26 levels and DPPIV enzyme activity appear to be useful biomarkers in patients with MPM. To further clarify the serum sCD26 levels and DPPIV enzyme activity in patients with MPM, we next analyzed the serum sCD26 levels and DPPIV enzyme activity among patients with MPM according to clinical stage. The serum sCD26 levels were significantly decreased in advanced stages (stage III and IV) compared with earlier stages (stage I and II) (P = 0.047, Fig. 3A), whereas there was no difference in DPPIV enzyme activity according to the clinical stage of MPM (P = 0.333, Fig. 3B). Next, we determined the association between the levels of sCD26 or DPPIV enzyme activity and the OS of patients with MPM. As shown in Fig. 3C, the median OS of patients with MPM who had higher DPPIV enzyme activity (≥17.0 µM/min) was 15.0 months (95% CI, 8.1–21.9 months), which was significantly longer than that of those with lower DPPIV enzyme activity (<17.0 µM/min) who had a median OS of 11.4 months (95% CI, 7.8–15.0 months) (P = 0.032, log-rank test). Meanwhile, there was no difference in OS between patients with higher (≥1.00 µg/ml) and lower (<1.00 µg/ml) sCD26 levels (Fig. 3D, P = 0.660, log-rank test). These data strongly suggest that serum levels of DPPIV enzyme activity are a predictive biomarker for the prognosis of patients with MPM.

**Figure 4:**
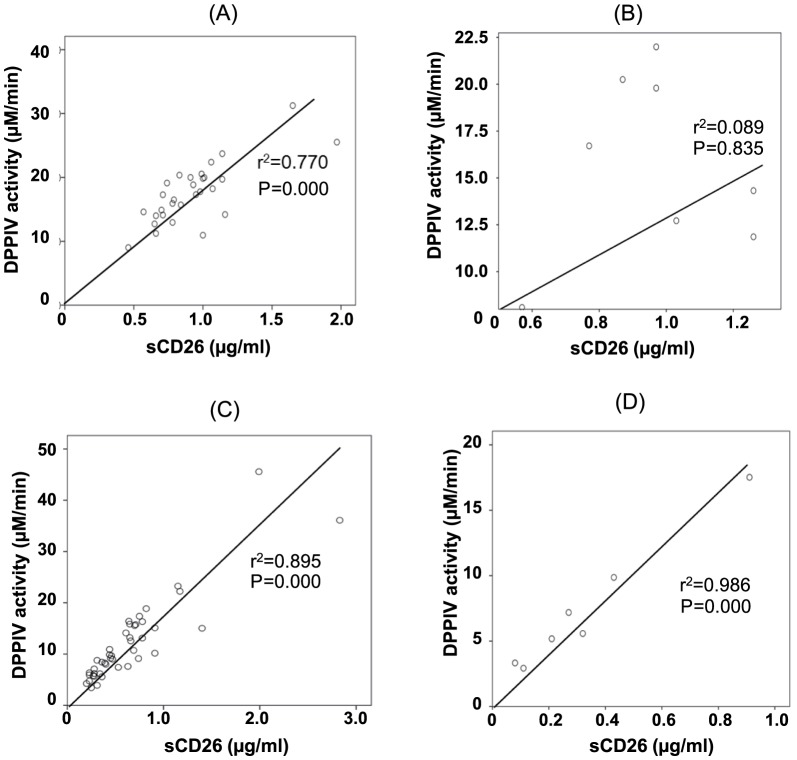
Correlation between sCD26 levels and DPPIV enzyme activity. Correlation between sCD26 levels and DPPIV enzyme activity in the serum of patients with (A) an epithelioid subtype and (B) sarcomatous subtype of MPM; and in the pleural fluid of patients with (C) an epithelioid subtype and (D) a sarcomatous subtype of MPM.

Next, we examined the correlation between DPPIV enzyme activity and sCD26 in serum from patients with MPM. Serum DPPIV enzyme activity was correlated with sCD26 in patients with an epithelioid subtype (r^2^ = 0.770, P = 0.000, Fig. 4A), but not in patients with a sarcomatous subtype (r^2^ = 0.089, P = 0.835, Fig. 4B).

### sCD26 and DPPIV Activity in the Pleural Fluid of Patients with MPM

**Figure 5:**
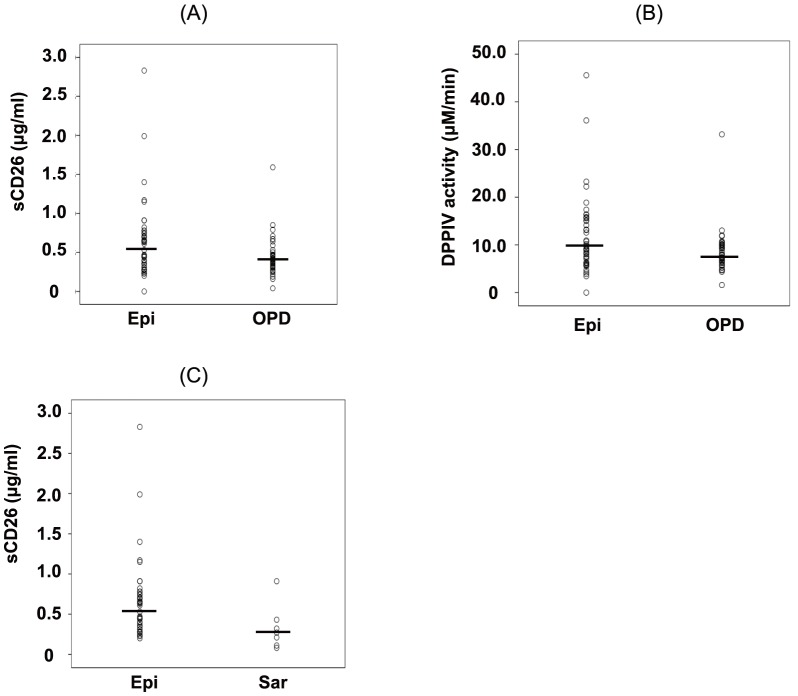
The levels of sCD26 or DPPIV enzyme activity in pleural fluid. (A) sCD26 levels and (B) DPPIV enzyme activity in the pleural fluid of patients with an epithelioid subtype of MPM (Epi) or with other pleural diseases (OPD). (C) sCD26 levels in the pleural fluid of patients with an Epi or sarcomatous (Sar) subtype of MPM. Each dot indicates an individual value and the horizontal bars indicate the median value.

To further determine the usefulness of sCD26 levels or DPPIV enzyme activity in patients with MPM, we assayed the levels of sCD26 or DPPIV enzyme activity in pleural fluid specimens from patients with MPM. DPPIV enzyme activity in pleural fluid was well correlated with sCD26 in both the epithelioid (r^2^ = 0.895, P = 0.000, Fig. 4C) and sarcomatous subtypes (r^2^ = 0.986, P = 0.000, Fig. 4D). As shown in Fig. 5A, sCD26 levels in the pleural fluid of MPM patients with an epithelioid subtype were significantly increased compared with the OPD cohort (P = 0.012). Moreover, DPPIV enzyme activity in the pleural fluid of MPM patients with an epithelioid subtype was significantly increased compared with that of the OPD cohort (P = 0.009, Fig. 5B). These results suggest that sCD26 levels or DPPIV enzyme activity may be good candidates as biomarkers in the pleural fluid of MPM patients with an epithelioid subtype.

To further clarify the role of sCD26 levels or DPPIV enzyme activity in pleural fluid, we analyzed the levels of sCD26 or DPPIV enzyme activity in the pleural fluid of MPM patients among patients with each histological subtype. As shown in Fig. 5C, sCD26 levels in the pleural fluid of MPM patients were significantly increased in patients with an epithelioid subtype compared with those with a sarcomatous subtype (P = 0.040). In addition, DPPIV enzyme activity in the pleural fluid of MPM patients tended to be increased in patients with an epithelioid subtype compared with those with a sarcomatous subtype (P = 0.077). These results suggest that sCD26 levels or DPPIV enzyme activity could be biomarkers in patients with an epithelioid subtype of MPM.

**Figure 6:**
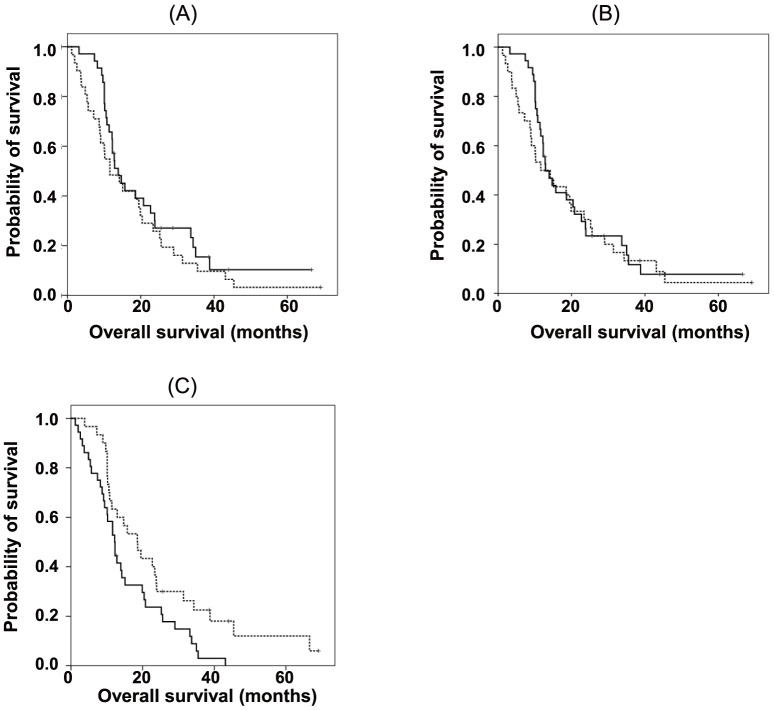
Overall survival in patients with malignant pleural mesothelioma according to soluble sCD26 levels. OS according to those with (A) higher (≥0.45 µg/ml, solid line) and lower (<0.45 µg/ml, dashed line) pleural fluid soluble sCD26 (sCD26) values; (B) higher (≥9.0 µM/min, solid line) and lower (<9.0 µM/min, dashed line) pleural fluid DPPIV enzyme activity; and (C) a higher (≥21.0, solid line) and lower (<21.0, dashed line) fraction of DPPIV/sCD26 in the pleural fluid.

To further determine the possibility that sCD26 levels or DPPIV activity in pleural fluid could act as a biomarker, we analyzed the OS of patients with MPM according to pleural fluid levels of sCD26 or DPPIV enzyme activity. Although we did not find a significant difference in OS according to sCD26 levels (P = 0.260) or DPPIV enzyme activity (P = 0.582) (Fig. 6A or B, respectively), patients with MPM who had a lower specific DPPIV activity, determined as DPPIV/sCD26 (<21.0 nmol/min/mg sCD26), had significantly prolonged survival compared with those with higher specific DPPIV activity (≥21.0 nmol/min/mg sCD26) (median OS: 18.5 months vs 12.2 months, P = 0.028 by log-rank test) (Fig. 6C). Taken together with the above data, our results strongly suggest that DPPIV activity in serum or pleural fluid may be a useful biomarker predictive of the prognosis of MPM patients.

#### Serum and pleural fluid SMRP

To make a comparative review of the usefulness of sCD26, we determined serum and pleural fluid SMRP. Median values of serum and pleural fluid SMRP in MPM patients were 0.43 and 15.37 mmol/l, respectively. Median value of pleural fluid SMRP in epithelioid MPM was 17.28 mmol/l. Median values of serum SMRP in SPE and pleural fluid SMRP in OPD were 0.90 and 0.43 mmol/l, respectively. Pleural fluid SMRP in MPM was significantly higher than in OPD (P = 0.000) and serum SMRP in MPM was significantly higher than in SPE (P = 0.000). To further clarify the usefulness of serum SMRP for differentiating MPM from SPE, we performed a ROC analysis. The AUC value for the differential diagnosis between these 2 groups was 0.738 (95% CI, 0.638–0.838) (data not shown).

## Discussion

We examined the usefulness of serum and pleural fluid sCD26 levels and DPPIV enzyme activity as clinical biomarkers of MPM. Serum sCD26 level and DPPIV enzyme activity were significantly decreased in patients with MPM compared with the SPE group. Generally, negative biomarkers have been difficult as markers of clinical significance. However, these results indicate the usefulness of these markers for early detection of MPM among the SPE group. Our results indicate that sCD26 could be compared favorably with SMRP, which is one of the most promising molecular biomarker of MPM at this time. In addition, sCD26 and DPPIV enzyme activity in pleural fluid was increased in patients with an epithelial subtype of MPM, and higher than those with OPD. These results indicate the clinical significance of sCD26 levels and DPPIV enzyme activity in pleural fluid as a diagnostic marker of the epithelial subtype of MPM. Furthermore, survival analyses demonstrated that serum DPPIV enzyme activity and specific DPPIV enzyme activity, determined as DPPIV/sCD26 in pleural fluid, could be a prognostic factor in patients with MPM.

MPM cases are usually diagnosed at an advanced stage and show poor response to treatment, so it is important to establish a molecular biomarker that can help diagnose MPM at earlier stages. In addition, focus should be put on screening high-risk subjects because most cases of MPM develop among those with an occupational or environmental history of past asbestos exposure. In this regard, some previous reports of molecular diagnostic markers for MPM exist. Robinson et al reported that serum SMRP was higher in patients with MPM compared with those with other cancers or other inflammatory lung or pleural diseases [24]. They also reported that 7 of 40 asbestos-exposed individuals had elevated serum concentrations of SMRP, and 3 of those 7 developed MPM within 5 years [24]. Scherpereel et al also reported that the serum SMRP level was higher in patients with MPM than in patients with pleural metastasis or benign pleural diseases [6]. Pass et al reported that serum osteopontin was higher in patients with MPM than in subjects with a history of asbestos exposure [25]. Recently, Shiomi et al reported that serum N-ERC/mesothelin levels were higher in patients with MPM compared with those with other diseases, including asbestos-related nonmalignant diseases [8]. In these previous reports, the definition of the control group was ambiguous; some included healthy subjects with a history of asbestos exposure, whereas others included patients with other asbestos-related benign diseases such as asbestosis. In the current study, serum sCD26 levels in patients with MPM were compared with those with past asbestos exposure and pleural plaques.

Pleural plaques are discrete, white to yellow-white, irregularly shaped, frequently calcified, and raised structures involving the parietal pleura [26]. They are not included in asbestos-related pleural diseases, but are established as a medical indicator of past asbestos exposure. Future studies are warranted to compare the utility of these markers for the differential diagnosis of MPM with a unified control group. In addition, the combination of these markers should be examined for a more accurate differential diagnosis.

There are some previous reports concerning the significance of CD26 levels in malignant conditions. Previous studies of CD26 have yielded varying results in different cancers. Preclinical studies show that increased CD26 expression inhibited metastasis in ovarian cancer [27], whereas suppression of CD26 promoted metastasis in prostate cancer [28]. On the other hand, inhibition of CD26 in renal cell carcinoma decreased tumor growth and reduced the ability of cancer cells to bind to fibronectin and collagen [17]. Moreover, clinical studies in thyroid cancer, gastrointestinal stromal tumors, and T cell non-Hodgkin's lymphoma/leukemias suggested that CD26 expression was associated with distant metastasis, recurrence after resection, or poor survival [29]–[31]. The multiple functions of CD26 may account for its various roles in different cancers [32]. Our recent study showed that CD26 expression in mesothelioma cells was associated with enhanced proliferative activity [19], and that CD26-positive mesothelioma cell lines appeared to have the characteristics of cancer stem cells [33].

The current study demonstrated that serum sCD26 levels were decreased in patients with MPM. Previously, Cordero et al reported that serum sCD26 was significantly lower in patients with colorectal cancer compared with healthy donors [34]. Their results are similar to those in the current study in terms of serum sCD26 levels being lower in cancer patients. As Cordero described, these findings indicate that the drop in sCD26 levels are related to an impaired immune system. These speculations are supported by data showing that CD26 and DPPIV activity are critical in T-cell biology as markers of T-cell activation. In addition, our current study demonstrated that serum sCD26 levels were decreased in advanced stages of MPM. Based on these findings, serum sCD26 levels might reflect impaired immune functions during the development and progression of MPM. Alternatively, there is another recent perception that serum DPPIV activity is one of the so-called adipokines, which are produced and released from adipose tissue [35], [36]. These adipokines are increased in obesity and reduced after weight loss, and are potential biomarkers of metabolic syndrome [37]. The relationship between decreased sCD26 and weight loss due to the development or progression of MPM should be clarified in future investigations.

In the current study, we also determined the sCD26 levels in the pleural fluid of patients with MPM and showed that sCD26 levels were higher in patients with an epithelioid subtype of MPM compared with those with a sarcomatous subtype. In a recent report, we demonstrated that CD26 expression in the tumor was higher in the epithelioid subtype of MPM than in other subtypes [19]. Based on these results, we suggest that sCD26 levels in the pleural fluid is secreted or released from MPM cells in the thorax. All these findings indicate that sCD26 in the serum and pleural fluid is released by different mechanisms. These findings are quite interesting in terms of the significance of CD26 levels in patients with MPM, in addition to the clinical usefulness of sCD26 as a molecular biomarker.

In previous reports, DPPIV enzyme activity was correlated with sCD26 concentration in healthy subjects and patients with type II diabetes [23], [38]. In the current study, the correlation was shown in patients with MPM except for those with the sarcomatous subtype (Fig. 4B). Although we cannot exclude the possibility that any genetic mutations or epigenetic modifications in CD26 appear to occur and potentiate DPPIV enzyme activity in the sarcomatous subtype of MPM, the discrepancy of specific DPPIV enzyme activity in the serum of patients with a sarcomatous subtype of MPM will be investigated in future studies.

In conclusion, we demonstrate the clinical significance of sCD26 levels and DPPIV activity in the sera and pleural fluid of patients with MPM. Serum sCD26 levels or DPPIV enzyme activity might be useful as early diagnostic markers or prognostic markers in patients with MPM, or as a screening tool to detect those at high-risk for development of MPM among SPE. The sCD26 levels in pleural fluid could be a useful diagnostic marker of the epithelioid subtype of MPM. Further validation studies are essential to clarify the clinical usefulness of sCD26 levels in patients with MPM.

## Supporting Information

S1 Table **Soluble CD26 (sCD26) levels and dipeptidyl peptidase IV (DPPIV) enzyme activity values.** The median and interquartile range values of the serum and pleural fluid sCD26 levels and DPPIV enzyme activity are shown.
(DOCX)
