# Supplementary material for: Clinical Significance of Soluble CD26 in Malignant Pleural Mesothelioma
Source: PLoS One. 2014 Dec 19;9(12):e115647. doi: 10.1371/journal.pone.0115647 (PMC4272261; doi:10.1371/journal.pone.0115647)
Supplement: S1 Table — Soluble CD26 (sCD26) levels and dipeptidyl peptidase IV (DPPIV) enzyme activity values. The median and interquartile range values of the serum and pleural fluid sCD26 levels and DPPIV enzyme activity are shown. (DOCX) [file pone.0115647.s001.docx]

**Table S1** Soluble CD26 (sCD26) levels and dipeptidyl peptidase IV (DPPIV) enzyme activity values.

| **Material** | | **Serum^a^** | | | **Pleural fluid^a^** | | |
| --- | --- | --- | --- | --- | --- | --- | --- |
| **Item** | | **sCD26** | **DPPIV** | **DPPIV/sCD26** | **sCD26** | **DPPIV** | **DPPIV/sCD26** |
| **Unit** | | **μg/ml** | **μM/min** | **nmol/min/mg sCD26** | **μg/ml** | **μM/min** | **nmol/min/mg sCD26** |
| **MPM** | **Total** | 0.89 (0.71-1.03) | 16.81 (12.96-19.83) | 19.79 (14.19-21.17) | 0.47 (0.3-0.71) | 9.17 (6.21-14.13) | 21.51 (18.94-24.39) |
|  | **I,II** | 0.98 (0.78-1.07) | 17.89 (15.71-20.35) | 18.20 (13.88-23.36) | 0.53 (0.36-0.72) | 9.67 (7.41-14.13) | 18.94 (13.88-23.36) |
|  | **III,IV** | 0.79 (0.78-1.07) | 14.76 (14.01-16.50) | 20.47 (19.28-25.82) | 0.40 (0.28-0.56) | 8.19 (7.41-14.13) | 22.10 (19.28-25.82) |
|  | **Epi** | 0.91 (0.71-1.00) | 17.32 (14.12-19.83) | 19.81 (18.16-21.17) | 0.53 (0.31-0.74) | 9.67 (6.21-15.12) | 20.72 (16.63-22.98) |
|  | **Bip** | 0.75 (0.70-0.79) | 12.68 (9.15-16.22) | 11.42 (9.69-13.16) | 0.50 (0.32-0.56) | 11.23 (7.70-12.92) | 23.26 (19.92-26.24) |
|  | **Sar** | 0.97 (0.77-1.03) | 15.52 (11.86-19.79) | 17.34 (11.38-22.62) | 0.27 (0.11-0.43) | 5.58 (3.33-9.87) | 24.21 (19.28-27.90) |
| **OPD** | | - | - | - | 0.42 (0.31-0.57) | 7.73 (5.66-10.09) | 20.01 (15.08-22.61) |
| **SPE** | | 1.14 (0.98-1.33) | 21.29 (18.07-24.27) | 18.01 (16.49-20.02) | - | - | - |

Epi: epithelioid, Bip: biphasic, Sar: sarcomatous, MPM: malignant pleural mesothelioma, OPD: other pleural diseases, SPE: subjects with past asbestos exposure.

^a^Each value represents the median and interquartile range.
